# The spontaneous mutation rate of *Drosophila pseudoobscura*

**DOI:** 10.1093/g3journal/jkab151

**Published:** 2021-05-05

**Authors:** Marc Krasovec

**Affiliations:** CNRS, Biologie Intégrative des Organismes Marins (BIOM), Observatoire Océanologique, Banyuls-sur-Mer 66650, France

**Keywords:** mutation rate, spontaneous mutation, *Drosophila pseudoobscura*, hybrids

## Abstract

The spontaneous mutation rate is a very variable trait that is subject to drift, selection and is sometimes highly plastic. Consequently, its variation between close species, or even between populations from the same species, can be very large. Here, I estimated the spontaneous mutation rate of *Drosophila pseudoobscura* and *Drosophila persimilis* crosses to explore the mutation rate variation within the *Drosophila* genus. All mutation rate estimations in *Drosophila* varied fourfold, probably explained by the sensitivity of the mutation rate to environmental and experimental conditions. Moreover, I found a very high mutation rate in the hybrid cross between *D. pseudoobscura* and *D. persimilis*, in agreement with known elevated mutation rate in hybrids. This mutation rate increase can be explained by heterozygosity and fitness decrease effects in hybrids.

## Introduction

The spontaneous mutation rate, denoted *µ*, currently has been estimated in more than 60 species [Figure 1 of Krasovec *et al.* (2019)] from bacteria and eukaryotes (Lynch *et al.* 2016; Katju and Bergthorsson 2018), and recently in one archaeal species (Kucukyildirim *et al.* 2020). These studies pointed out a substantial variation in the mutation rate at all scales. One of the most interesting findings is the large variation of *µ* between close species, or between strains or populations from a same species. One of the most cited studies is the case of the green alga *Chlamydomonas reinhardtii* where *µ* varies sevenfold between several strains (Ness *et al.* 2015). More recently, a study based on a mutation accumulation experiment of three populations of *Daphnia magna* reported an even greater variation in *µ*, from 3.6 × 10^−9^ to 3.4 × 10^−8^ (Ho *et al.* 2020). A putative explanation of this variation is the effect of the environment on the mutation rate, which has been reported in yeast (Liu and Zhang 2019), *Escherichia coli* (Chu *et al.* 2018), and *Arabidopsis thaliana* (Jiang *et al.* 2014). For instance, in *A. thaliana*, stress seems to be at the origin of mutation rate increase, which was twice as high in stressful than in benign conditions. It so appears that the mutation rate is a plastic trait and its estimation can vary between experimental conditions. This raises questions about the repeatability of mutation accumulation experiments in the same species, addressed in a previous study (Behringer and Hall 2016) showing that *µ* estimated from independent experiments are significantly different in *Drosophila melanogaster*, *Caenorhabditis elegans*, and *Schizosaccharomyces pombe.* In *D. melanogaster*, for example, a significant approximately twofold variation of mutation rate (Welch’s *t*-test, *P*-value < 0.05) has been reported by Behringer and Hall (2016) using previous data (Schrider *et al.* 2013; Keightley *et al.* 2014).

If environment or culture conditions have a high impact on the mutation rate, then we can reasonably expect that the mutation rate estimated under laboratory conditions may be far from the real mutation rate in the wild. This is also relevant for closely related species or strains, which should have different mutation rates as a function of their ecological and environmental ranges even with a similar genomic background. On the other hand, there are several reported cases where the mutation rate estimated from close species are similar such as in the *Caenorhabditis* genus (Denver *et al.* 2012); or much lower than observed in *C. reinhardtii* (Ness *et al.* 2015) and *D. magna* (Ho *et al.* 2020), such as in *Mycobacterium* (Ford *et al.* 2011; Kucukyildirim *et al.* 2016) or *Vibrio* (Dillon *et al.* 2016; Strauss *et al.* 2017) genera. In this study, I took advantage of available parents-progenies genomic data from a previous study to measure the mutation rate of *Drosophila pseudoobscura* and *Drosophila persimilis* crosses (Korunes and Noor 2019) and explore the mutation rate variation within *Drosophila* genus.

## Materials and methods

Genomic data analyzed were from a previous study on *Drosophila* crosses (Korunes and Noor 2019). Briefly, Korunes and Noor did three crosses to F2 generations with three genotypes of *D. pseudoobscura* and one genotype of *D. persimilis* [see Figure 2 of Korunes and Noor (2019)]. A grandparent female *D. pseudoobscura* PP1137 (isolated in New Mexico, USA) was crossed with three males *D. pseudoobscura* PP1134 (isolated in New Mexico, USA), *D. pseudoobscura* MSH177 (isolated in California, USA), and *D. persimilis* MSH1993 (isolated in California, USA), which gave the three F1 parent generations. Then, a female from this F1 generation was crossed with a *D. pseudoobscura* PP1137 male, which gave the three F2 progeny generations. All genomes (2 grandparents, 2 F1 parents, and 10 F2 progenies for each cross) were sequenced by Illumina HiSeq 100-bp paired-end with 20× coverage (SRR numbers provided in Supplementary Table S1). A summary of the crosses is provided in Supplementary Figure S1.

The mutation identification was done following the highly stringent method developed previously (Schrider *et al.* 2013; Keightley *et al.* 2014) that gave almost 100% of true positives. Raw reads were aligned against the *D. pseudoobscura* PP1137 reference genome with bwa mem (Li and Durbin 2010), then the resulting bam files were treated with samtools (Li *et al.* 2009), and SNP calling was done with HaplotypeCaller from GATK (McKenna *et al.* 2010). Mutation calling was done separately for each cross. A candidate mutation was considered true only if it fulfilled the following conditions: the site was covered by at least 10 reads in all individuals (the 2 grandparents, the 2 F1 parents, and the 10 F2); all sites with at least one individual with 50 coverage or more were discarded to limit false positives due to repetitive elements; the mutation is identified in only one F2 individual; the site was strictly homozygous in all individuals without any alternative allele, even if support by only one read; the alternative allele had a coverage of five or more and was supported by at least 20% of total site coverage; all candidate sites were verified by visual inspection of the mpileup files (alignment text files generated by samtools) and with IGV (reads with more than 2% mismatches were considered as miss mapping) (Robinson *et al.* 2011); and all mutation candidates inside an indel were removed with bcftools, option -snpgap 1 (Li 2011). Last, final mutation candidates were checked in other crosses, even if all individuals did not have coverage of 10 or more. The mutation rate was calculated counting one generation for the F2 because parents and grandparents were used as ancestral lines: $µ=\;\frac{N}{G*2*n*g}\;$where *N* is the number of mutations, G* the number of callable sites (G*2 because the genome is diploid and *µ* is calculated per site per haploid genome), *n* the number of F2 progenies and *g* the number of generations (here *g* = 1).

### Data availability

Raw reads data of the crosses are available under bioproject PRJNA492790. Reference *D. pseudoobscura* and *D. persimilis* raw reads are submitted in the SRA database under numbers SRR330107 and SRR363439, respectively. Supplementary files are available on the G3 figshare portal: https://doi.org/10.25387/g3.14501343. Supplementary Table S1 containing SRA accession numbers of raw reads used in this study; Supplementary Figure S1 summarizing the three *Drosophila* crosses; File IGV_denovo _screenshots containing IGV screen shots of mutated positions.

## Results and discussion

In all, I identified 14 nucleotide mutations in 28 F2 progenies. Two F2 progenies were removed because of low coverage, one from the cross PP1134 and one from the cross MSH1993. No mutations were found in the cross PP1134, 4 mutations in the cross MSH177, and 10 mutations in the cross MSH1993. On the 15 identified mutations within the cross MSH1993, 9 arose in a same individual. The mutations were GC -> AT biased, with an AT bias of 3.7 and 12.2 in the crosses MSH177 and MSH1993 (assuming one hypothetical AT to GC mutation in MSH1993 cross), respectively. This GC -> AT bias is very is common in metazoans and even in all eukaryotes [see Table 2 of Katju and Bergthorsson (2018), Supplementary Table S11 of Krasovec *et al.* (2017)]. Only one reported eukaryote species has a GC -> AT mutation bias, the haptophyte *Emiliania huxleyi* (Krasovec *et al.* 2020).

**Table 2:** Identified mutations in the three crosses

Crosses	N_F2_	G*	Mutations (contig; position; reference; mutation)			
*D. pseudoobscura* PP1137 × *D. pseudoobscura* PP1134	9	20,329,686	No mutation			
*D. pseudoobscura* PP1137 × *D. pseudoobscura* MSH177	9	63,534,699	NC_046680.1	17833723	G	A
			NC_046681.1	6119653	C	T
			NC_046681.1	1779949	C	A
			NC_046679.1	918510	T	C
*D. pseudoobscura* PP1137 × *D. persimilis* MSH1993	10	25,855,578	NC_046680.1	21310719	G	T
			NC_046680.1	13587576	G	T
			NC_046681.1	19040615	C	A
			NC_046681.1	20681630	G	T
			NC_046679.1	2025226	C	A
			NC_046679.1	21427501	T	A
			NC_046679.1	23593472	C	A
			NC_046680.1	11444083	G	T
			NC_046681.1	6516206	G	T
			NC_046681.1	26994424	C	A

*N_F2_* is the number of F2 progenies, G* is the number of callable sites.

The calculated mutation rates are *µ* <2.7 × 10^−09^ for the cross PP1134 assuming one hypothetical mutation, *µ* = 3.5 × 10^−09^ (Poisson CI: 0.7 × 10^−09^—10.2 × 10^−09^) for the cross MSH177 and *µ* = 19.3 × 10^−09^ (Poisson CI: 9.3 × 10^−09^—35.6 × 10^−09^) for the cross MSH1993 (Table 1). However, the *Drosophila* genome is about 165 Mb and the callable genome for the three crosses was 20, 26, and 63 Mb (Table 2). The callable genome size of the cross MSH1993 is probably reduced due to the divergence between the two species with an average nucleotide identity of 97.7% estimated with fastANI (Jain *et al.* 2018). Mutation candidates due to miss mapping were removed by IGV inspection (see *Materials and methods*) and IGV screenshots of *de novo* mutations are provided in supplementary documentation. Considering the variation of the mutation rate within a genome, we cannot exclude that the mutation rates estimated here do not reflect the average mutation rate. The mutation rate varied by approximately fourfold between the lower and the higher measures in the *Drosophila* genus (Table 1), excluding the cross MSH1993 that is a particular case of hybrid species (see below). Taking the cross MSH1993 into account, this variation reached ∼11-fold, which is the same mutation rate variation across metazoan species that counts several estimations from mammals (Uchimura *et al.* 2015; Besenbacher *et al.* 2016; Koch *et al.* 2019), birds (Smeds *et al.* 2016), worms (Denver *et al.* 2012; Weller *et al.* 2014), and arthropods (Schrider *et al.* 2013; Keightley *et al.* 2014; Flynn *et al.* 2017; Liu *et al.* 2017; Oppold and Pfenninger 2017).

**Table 1:** Spontaneous mutation rates per site per haploid genome in *Drosophila* genus

Species	*µ* (× 10^−9^)	Ref
*D. melanogaster* (Massachusetts, USA)	5.5	Schrider *et al.* (2013)
*D. melanogaster* (Ghana)	2.8	Keightley *et al.* (2014)
*D. melanogaster* DGRP360 (North Carolina, USA)	5.2	Huang *et al.* (2016)
*D. melanogaster* (Madrid line)	3.5	Keightley *et al.* (2009)
*D. melanogaster* DGRP765 (North Carolina, USA)	4.9	Assaf *et al.* (2017)
*D. melanogaster* (North Carolina, USA)	6.0	Sharp and Agrawal (2016)
*D. melanogaster* (Madrid, Spain)	2.7	Haag-Liautard *et al.* (2007)
*D. melanogaster* Florida-33 (Florida, USA)	11.7	
*D. melanogaster* Florida-39 (Florida, USA)	6.8	
*D. pseudoobscura* PP1137 × *D. pseudoobscura* PP1134	<2.7^a^	This study
*D. pseudoobscura* PP1137 × *D. pseudoobscura* MSH177	3.5	
*D. pseudoobscura* PP1137 × *D. persimilis* MSH1993	19.3	

The mutation rates between MSH177 and MSH1993 (hybrids) crosses are significantly different (Chi-squared test, *P*-value = 0.003).

a Mutation rate corresponding to 1 mutation.

Mutation rate variation within the same species, or between close species, is starting to be well documented (Ness *et al.* 2015; Behringer and Hall 2016; Chu *et al.* 2018; Liu and Zhang 2019; Ho *et al.* 2020). The main explanation of such variation is the effect of the environment on the mutation rate. The different isolated *Drosophila* lines used in this study came from different places in United States, Spain, and Africa, corresponding to variable ecological range. Given the sensitivity of the mutation rate to the environment, it is possible that these lines evolved several different mechanisms that control mutation rate or dedicate different amounts of energy to control it. Also, the different experimental conditions that the lines were subjected to between the published studies may have directly increased or decreased the mutation rate. Specifically, experimental conditions will influence factors such as metabolic rate, generation time, oxidative stress or energy devoted to replication/fidelity, and mutagen control, all of which impact the mutation rate (Martin and Palumbi 1993; Baer *et al.* 2007). Oxidative stress, which is linked to the metabolic rate, has been known for years to increase the mutation rate (Dizdaroglu 1992; Cooke *et al.* 2003), particularly via guanine oxidation. The generation time is also a key parameter, particularly in multicellular species, because it defines the number of germline cell divisions and the time where the germinal lines are subjected to any mutagenic process. In primates, the spontaneous mutation rate decreases when the generation time is shorter (Thomas *et al.* 2018; Wu *et al.* 2020).

The case of the cross MSH1993 is a particular because it is a cross between two species, *D. pseudoobscura* and *D. persimilis*. Hybrids often have higher mutation rate than initial species, such as was reported in *Drosophila* many years ago (Belgovsky 1937; Sturtevant 1939), and more recently in plants from the *Prunus* genus (Xie *et al.* 2016). However, here we do not have the mutation rate of *D. persimilis*. We so cannot exclude that *D. persimilis* may have a particularly high mutation rate compared to other *Drosophila* species, increasing the mutation rate of the hybrid cross. The mutation rate of the hybrid cross is, however, much higher than in any other *Drosophila* study and of the same order of higher mutation rate ever measured in the tree of life (Katju and Bergthorsson 2018). In *Arabidopsis*, it is hybrids between populations or ecotypes that induce a mutation rate change (Bashir *et al.* 2014). The reasons for this observed increase of mutation rate in hybrids remains unclear, although there are several proposed explanations. First, the heterozygosity associated mutation rate is the observation that heterozygosity is positively linked to the mutation rate (Amos 2010; Yang *et al.* 2015). In that case, it is proposed that the higher heterozygosity in hybrids compared to original species induces an elevated mutation rate. Another explanation is the possible fitness decrease of the hybrids compared to original species. Stress or fitness loss is known to be at the origin of mutation rate increase in *Arabidopsis* and *Drosophila* (Baer 2008; Sharp and Agrawal 2012; Jiang *et al.* 2014) and thus may similarly be the main contributor to higher mutation rate in hybrids.
